# Vitamin D deficiency osteomalacia triggered by long-term social withdrawal and unbalanced diet in a Japanese middle-aged subject: A case report

**DOI:** 10.1097/MD.0000000000028589

**Published:** 2022-01-14

**Authors:** Yuichiro Iwamoto, Fuminori Tatsumi, Kazunori Dan, Yukino Katakura, Masashi Shimoda, Tomohiko Kimura, Shuhei Nakanishi, Tomoatsu Mune, Kohei Kaku, Hideaki Kaneto

**Affiliations:** a Department of Diabetes, Endocrinology and Metabolism, Kawasaki Medical School, Kurashiki, Japan; b Kawasaki Medical School General Medical Center, Okayama, Japan.

**Keywords:** atopic dermatitis, gait disturbance, hypophosphatemia, social withdrawal, vitamin D deficiency osteomalacia

## Abstract

**Introduction:**

Osteomalacia is caused by an increase in the number of osteoids owing to mineralization failure. There are various causes of osteomalacia, such as hypophosphatemia due to excess production of fibroblast growth factor 23, vitamin D deficiency, insufficient vitamin D action, and renal tubular disorders.

**Patient concerns:**

A 53-year-old man with bone pain and gait disturbance was referred to our institution. At the age of 35, he developed atopic dermatitis. He had eyesight deterioration due to atopic cataracts when he was 37 years old. Subsequently, he stayed home all the time, and his eating habits were unbalanced for a long period of time. Although he had atopic dermatitis, he did not take allergen-free diets, and he did not use sunscreen. Furthermore, when he was 43 years old, he failed to flex his legs and suffered gait disturbance.

**Diagnosis:**

Hypocalcemia and hypophosphatemia were observed as follow: calcium, 5.5 mg/dL; adjusted calcium, 6.9 mg/dL; inorganic phosphorous, 1.9 mg/dL. In addition, intact parathyroid hormone levels were as high as 277.4 pg/mL, and 1, 25-(OH)_2_ vitamin D and 25-(OH) vitamin D levels were markedly reduced: 1, 25-(OH)_2_ vitamin D, ≤4 pg/mL; 25-(OH) vitamin D, 11.0 ng/mL. Fibroblast growth factor 23 levels did not increase. Alkaline phosphatase (ALP) and bone-type ALP (BAP) levels were high: ALP, 784 U/L; BAP, 159.2 μg/L (reference range: 3.7–20.9 μg/L). Based on these findings, we diagnosed this patient with vitamin D-deficient osteomalacia triggered by long-term social withdrawal and an unbalanced diet.

**Interventions and outcomes:**

After hospitalization, to treat vitamin D-deficient osteomalacia, we started to administer 1 μg/day of alfacalcidol and 3 g/day of calcium lactate. Approximately one month later, 1,25-(OH)_2_ vitamin D levels increased to 214 pg/mL. Consequently, calcium and inorganic phosphorus were also increased up to 7.8 mg/dL and 3.9 mg/dL, respectively, and intact parathyroid hormone was decreased to 132.0 pg/mL.

**Conclusions:**

We should bear in mind the possibility of osteomalacia triggered by social withdrawal and vitamin D deficiency even in middle-aged subjects.

## 1 Introduction

Osteomalacia is a bone metabolic disorder whose main condition is bone mineralization failure and consequent increment of osteoids in adulthood and childhood. Mineralization of the bone matrix with the deposition of calcium phosphate or calcium carbonate is very important for the formation of normal bone. Increment of the osteoid leads to bone pain and/or fracture. There are various causes of osteomalacia, such as hypophosphatemia due to excess production of fibroblast growth factor 23 (FGF 23), vitamin D deficiency and resistance, renal tubular acidosis, tumors, hypophosphatasia, or mineralization inhibitors.^[1–8]^ Osteomalacia, especially vitamin D-deficient osteomalacia, is rarely observed in midlife, at least in Japan, although it is common in certain countries, particularly in southern Asia and Europe.

Vitamin D increases calcium (Ca) and phosphorous levels by increasing the reabsorption of Ca and phosphorous in the renal tubules and in the small intestine. In addition, vitamin D regulates magnesium levels by increasing renal reabsorption. Furthermore, vitamin D suppresses parathyroid hormone (PTH) secretion. It is well known that vitamin D is important for bone development and that vitamin D deficiency can lead to a variety of problems such as rickets in children and osteomalacia, osteoporosis, and hypocalcemia in adults.

Here, we present a middle-aged subject with vitamin D-deficient osteomalacia triggered by long-term social withdrawal and an unbalanced diet. Osteomalacia can be brought about in house-bound subjects; however, to the best of our knowledge, osteomalacia in this subject was extremely severe, at least among Japanese subjects.

## 2 Case presentation

A 53-year-old Japanese man was admitted to another emergency room because of bone pain, limb contracture, and pressure ulcers. He developed atopic dermatitis 18 years previously, was socially withdrawn due to severe atopic cataracts, and did not spend any time out of doors for as long as 16 years. He was unable to walk for 10 years because of limb contracture. He was suspected to have osteoporosis by a previous doctor, but he had hypophosphatemia and hypocalcemia. To examine the cause of the symptoms and electrolyte abnormalities, the patient was transferred to our institution.

On admission, his height and body weight were 165 cm and 39.7 kg (body mass index 14.6 kg/m^2^, indicative of malnutrition). His blood pressure, heart rate, and body temperature were 119/67 mm Hg, 112/min and 36.3°C. Table 1 shows the data on admission in this study. Liver and renal function were within normal range as follows: AST, 28 U/L; ALT, 16 U/L; γ-GTP, 13 U/L; creatinine, 0.29 mg/dL; BUN, 11.1 mg/dL. Alkaline phosphatase (ALP) and bone-specific ALP (BAP), a bone formation marker, were high: ALP, 784 U/L; BAP, 159.2 μg/L (reference range: 3.7–20.9 μg/L). In addition to the increase in BAP, both TRACP-5b, a bone resorption marker, and ucOC, a bone matrix-related marker, were substantially increased: TRACP-5b ≥1500 mU/dL (reference range: 170–590 mU/dL) and ucOC 148.20 ng/mL (0–4.5 ng/mL). Serum Ca and serum inorganic phosphorus (IP) were low: serum Ca, 5.5 mg/dL; adjusted serum Ca, 6.9 mg/dL; serum IP, 1.9 mg/dL. Vitamin D levels were markedly reduced: 1,25-(OH)_2_ vitamin D, <4 pg/mL, and 25-(OH) vitamin D, 11.0 ng/mL. There was no increase in FGF 23 levels, whereas intact PTH levels were as high as 277.4 pg/mL (reference range: 10–65 pg/mL). Anemia and hypoalbuminemia were observed (hemoglobin, 12.1 g/dL; albumin, 2.6 g/dL). Creatinine phosphokinase and C-reactive protein levels increased to 606 U/L and 3.95 mg/dL, respectively. In addition, we calculated the average estimated intake of Ca and vitamin D for 7 days based on an oral interview survey with this subject. The average estimated intakes of Ca and vitamin D were 370 mg and 5.7 μg, respectively, both of which were substantially lower than their estimated required amounts (600 mg and 8.5 μg, respectively). Furthermore, considering the fact that this subject stayed at home all the time and the synthesis of vitamin D precursors in the skin was not promoted by sunlight, it was likely that the required amounts of vitamin D from intake were much larger than this estimation in this subject.

**Table 1:** Clinical data on admission in this subject.

Peripheral blood	
Red blood cells	396 × 10^4^/μL
Hemoglobin	12.1 g/dL
Hematocrit	35.7%
White blood cells	9500/μL
Platelet	34.3 × 10^4^/μL
Endocrine markers	
TSH	1.025 μIU/mL
FT4	1.37 ng/dL
Intact PTH	277.4 pg/mL
1,25-(OH)_2_ VD	≤4 pg/mL
25-(OH) VD	11.0 ng/mL
FGF 23	<5.0 pg/mL
Blood biochemistry	
Total protein	7.3 g/dL
Albumin	2.6 g/dL
Total bilirubin	0.9 mg/dL
AST	28 U/L
ALT	16 U/L
γ-GTP	13 U/L
LDH	292 U/L
ALP	784 U/L
BAP	159.2 μg/L
Creatinine	0.29 mg/dL
BUN	11.1 mg/dL
Glucose	131 mg/dL
CK	606 U/L
CRP	3.95 mg/dL
Electrolytes	
Sodium	139 mEq/L
Potassium	3.5 mEq/L
Chloride	104 mEq/L
Calcium	5.5 mg/dL
Adjusted calcium	6.9 mg/dL
Phosphorous	1.9 mg/dL
Magnesium	1.5 mg/dL
Urinalysis	
Glucose	(−)
Protein	(±)
Occult blood	(−)
Phosphorous	4.4 mg/dL
%TRP	88.6%

%TRP = % tubular reabsorption of phosphate, γ-GTP = γ-glutamyl transpeptidase, ALP = alkaline phosphatase, ALT = alanine aminotransferase, AST = aspartate aminotransferase, BAP = bone-type alkaline phosphatase, BUN = blood urea nitrogen, CK = creatine phosphokinase, CRP = C-reactive protein, FGF 23 = fibroblast growth factor 23, FT4 = free thyroxine, LDH = lactate dehydrogenase, PTH = parathyroid hormone, TSH = thyroid-stimulating hormone, and VD = vitamin D.

**Figure 1:**
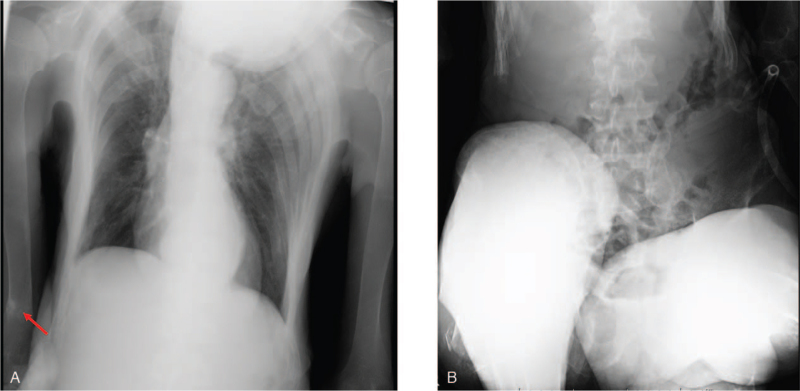
(A) In radiograph, there was looser's zone in humerus (a red arrow) which indicates low mineralization area and is characteristic for osteomalacia. (B) There was severe deformity in the long canal bone.

As shown in Figure 1A, Looser's zone in the right humerus was observed on radiography, which is indicative of osteomalacia. These findings are characteristic of osteomalacia. Also, as shown in Figure 1B, there severe deformities were observed in the long canal bone. The bone density was low; the percentage of femur mineral bone density compared to that in young adult men was 49% in dual-energy X-ray absorptiometry. Bone scintigraphy revealed multiple old fractures in the distal right arm and proximal bilateral femurs. Taken together, we diagnosed this subject with vitamin D-deficient osteomalacia associated with social withdrawal and an unbalanced diet.

After hospitalization, to treat vitamin D-deficient osteomalacia, we started administering 1 μg/day of alfacalcidol and 3 g/day of calcium lactate. Approximately 1 month later, 1,25-(OH)_2_ vitamin D levels increased to 214 pg/mL. Consequently, Ca and IP were also increased up to 7.8 mg/dL and 3.9 mg/dL, respectively, and intact PTH was decreased to 132.0 pg/mL. It is noted here that although natural vitamin D is promising for the treatment of vitamin D deficiency osteomalacia, it is not formally approved in Japan to use natural vitamin D for the treatment of vitamin D deficiency osteomalacia and thus synthesized active vitamin D alfacalcidol is often prescribed instead of natural vitamin D. Therefore, we used synthesized active vitamin D alfacalcidol during the period of hospitalization. Approximately two months later, the intact PTH level decreased to 87.0 pg/mL. Furthermore, bone pain was dramatically mitigated with these medications, and limb contracture was ameliorated with rehabilitation. Additionally, natural vitamin D is available as a food supplement in Japan. Therefore, we stopped the synthesized active vitamin D alfacalcidol on discharge (70 days after starting the treatment) and allowed him to take natural vitamin D as a supplement food after discharge. The serum Ca, IP, and intact PTH levels improved after discharge (Ca, 9.4 mg/dL; IP 5.0 mg/dL; intact PTH, 57 pg/mL). We failed to take Follow-up radiography was not performed because the lower limbs in this subject were fixed in the flexed position due to severe pain. However, we evaluated the CT values of the bones before and after the treatment. The average CT value in the fifth lumbar vertebral body increased from 149.1 to 257.3, and that in the femoral neck increased from 170.9 to 217.3. In addition, the percentage of femur mineral bone density compared with that in young adult men increased from 49% to 112% after treatment.

## 3 Discussion

Rickets and osteomalacia are pathological conditions caused by metabolic errors in bone mineralization. Tumor-induced osteomalacia often develops in adulthood, but other causes are relatively rare in Japan. Osteomalacia is classified into a subtype due to vitamin D deficiency and hypophosphatemia. According to the guidelines in Japan,^[1]^ vitamin D deficiency is diagnosed when serum 25-(OH) vitamin D levels are <20 ng/mL. Vitamin D deficiency osteomalacia is diagnosed by blood tests, including vitamin D deficiency, an elevation of serum ALP, hyperparathyroidism, and rickets change on radiographs. Based on these guidelines, the patient was diagnosed with vitamin D-deficient osteomalacia.

This subject stayed home all the time because of eye deterioration and became completely socially withdrawn for a long period of time. He also failed to flex his legs and suffered gait disturbances. Indeed, we failed to improve the quality of X-ray because the lower limbs in this subject were fixed in the flexed position due to severe pain. We believe that these phenomena also showed that the osteomalacia in this patient was extremely severe. In addition, he ate fairly unbalanced food for a long time. Indeed, 1,25-(OH)_2_ vitamin D and 25-(OH) vitamin D were both markedly decreased. There have been reports of cases of osteomalacia caused by food removal therapy in infants with allergic diseases,^[9]^ and the use of allergen-free diets and sunscreen in adults.^[10]^ In addition, vitamin D deficiency has been shown to be induced by reduced sun exposure due to atopic dermatitis. However, this was not the case for this study. Although he had atopic dermatitis, he did not take allergen-free diets and he did not use sunscreen. Based on these findings, we finally diagnosed this subject as having vitamin D-deficient osteomalacia triggered by long-term social withdrawal and an unbalanced diet. To the best of our knowledge, it is very rare for subjects in their 50 to suffer from this disorder.

Additionally, in Brazil, vitamin D-deficient osteomalacia was reported for the first time in a 62-year-old subject who had been misdiagnosed with osteoporosis for a long time. They mentioned that vitamin D-deficient osteomalacia in adulthood was frequently incorrectly diagnosed as osteoporosis.^[11]^ Our patient was initially misdiagnosed with osteoporosis. However, while subjects with osteoporosis were usually characterized by a normal range of Ca, IP, and ALP, this subject showed hypophosphatemia, hypocalcemia, and high ALP. In addition, Looser's zone was observed on radiographs of the right humerus, and vitamin D levels were very low in this patient. Therefore, we diagnosed this patient with vitamin D-deficient osteomalacia, although we failed to completely exclude the possibility that such osteomalacia was complicated with osteoporesis. The standard treatment for vitamin D-deficient osteomalacia is supplementation with natural vitamin D, whereas there are no prescriptionable formulations in Japan. Thus, it is desirable to prescribe active vitamin D3 preparations or supplements. This patient was administered an active vitamin D3 agent at our hospital, and he took supplemental supplements after discharge.

In general, primary hyperparathyroidism shows high PTH and Ca levels, but Ca levels were low in this subject, excluding the possibility of hyperparathyroidism. We think it is possible that hypocalcemia due to vitamin D deficiency increases PTH levels through negative feedback, leading to a decrease in IP. However, in this case, PTH levels were normalized to vitamin D treatment. These data suggest that high PTH levels are secondary alterations but not the main cause of pathology in this subject. In addition, there have been reports about the cause of vitamin D deficiency, such as inadequate intake of vitamin D due to long-term allergen-free diets and reduced sun exposure due to sunscreen, as the cause of osteomalacia associated with atopic dermatitis. However, this was not the case for this study. Although he had atopic dermatitis, he did not take allergen-free diets and he did not use sunscreen.

The main pathological conditions of vitamin D resistance are suppression of tubular reabsorption of phosphorous (%TRP) and decrease in blood 1,25-(OH)_2_ vitamin D due to overproduction of FGF 23.^[1–3]^ In this case, however, FGF 23 levels did not increase and %TRP was 86% (reference range, 85–95%), which ruled out this possibility. In adults, neoplastic osteomalacia or renal tubular disorders, such as Fanconi syndrome, could lead to the development of osteomalacia,^[1,4,5]^ with no findings indicating such diseases in this subject. Furthermore, TmP/GFR was as low as 1.65 mg/dL (reference range: 2.3–4.3 mg/dL) when IP was 1.9 mg/dL, indicating that phosphorus maximum tubular reabsorption was not increased even with low IP conditions in this subject. Therefore, although speculative, we assume that some tubular disorders are involved in such phenomena.

Vitamin D-dependent osteomalacia is a genetic disease induced by mutations in 25-(OH) vitamin D-1α-hydroxylase (type 1) or vitamin D receptor (type 2). Symptoms are usually observed several months after birth.^[1]^ In this subject, however, there was no problem with growth and development in his childhood, which excluded this possibility. In addition, considering the data with very low 1,25-(OH)_2_ vitamin D and 25-(OH) vitamin D levels, it is possible, at least in theory, that such vitamin D reduction is triggered by congenital CYP27R1 deficiency. However, in this subject, we think such a possibility is quite low for the following two reasons. First, there were no problems with growth and development in his childhood. Second, 1 of 25-(OH)_2_ were substantially increased after administration of the natural vitamin D preparation, in which the OH residue was not modified at all. Therefore, it is unlikely that there were any abnormalities in the OH modification system in this study.

Taken together, we should bear in mind the possibility of vitamin D-deficient osteomalacia even in middle-aged subjects, especially when hypocalcemia and hypophosphatemia with bone pain are observed in subjects with a history suggestive of long-term inadequate intake of vitamin D and long-term reduced sun exposure, such as social withdrawal. In addition, since vitamin D-deficient osteomalacia in adults is often misdiagnosed as osteoporosis, we should be very careful for such differential diagnosis in routine medical care.

## Author contributions

Y.I., F.T., and H.K. collected the data and wrote the manuscript. K.D., Y.K., M.S., T.K., S.N., T.M., and K.K. contributed to discussion.

**Data curation:** Yuichiro Iwamoto.

**Investigation:** Kazunori Dan, Yukino Katakura, Masashi Shimoda, Tomohiko Kimura, Shuhei Nakanishi, Tomoatsu Mune, Kohei Kaku.

**Writing – original draft:** Yuichiro Iwamoto.

**Writing – review & editing:** Fuminori Tatsumi, Hideaki Kaneto.
